# Ethnomedicinal Plant Knowledge of the Karen in Thailand

**DOI:** 10.3390/plants9070813

**Published:** 2020-06-29

**Authors:** Methee Phumthum, Henrik Balslev, Rapeeporn Kantasrila, Sukhumaabhorn Kaewsangsai, Angkhana Inta

**Affiliations:** 1 Department of Pharmaceutical Botany, Faculty of Pharmacy, Mahidol University, Bangkok 10400, Thailand; methee.phu@mahidol.edu; 2 Sireeruckhacharti Nature Learning Park, Mahidol University, Nakhon Pathom 73170, Thailand; 3 Department of Biology, Faculty of Natural Science, Aarhus University, 8000 Aarhus, Denmark; henrik.balslev@bios.au.dk; 4 Department of Biology, Faculty of Science, Chiang Mai University, Chiang Mai 50200, Thailand; rapeeporn_ka@cmu.ac.th (R.K.); sukhumaabhorn_kaew@cmu.ac.th (S.K.); 5 Research Center in Bioresources for Agriculture, Industry and Medicine, Chiang Mai University, Chiang Mai 50200, Thailand

**Keywords:** medicinal plants, traditional knowledge, ethnic group, ethnobotany, important plants

## Abstract

The Thai Karen, the largest hill-tribe in Thailand, guard substantial ethnomedicinal plant knowledge, as documented in several studies that targeted single villages. Here, we have compiled information from all the reliable and published sources to present a comprehensive overview of the Karen ethnomedicinal plant knowledge. Our dataset covers 31 Karen villages distributed over eight provinces in Thailand. We used the Cultural Importance Index (CI) to determine which species were the most valuable to the Karen and the Informant Consensus Factor (ICF) to evaluate how well distributed the knowledge of ethnomedicinal plants was in various medicinal use categories. In the 31 Karen villages, we found 3188 reports of ethnomedicinal plant uses of 732 species in 150 plant families. *Chromolaena odorata*, *Biancaea sappan*, and *Tinospora crispa* were the most important medicinal plants, with the highest CI values. The Leguminosae, Asteraceae, Zingiberaceae, Euphorbiaceae, Lamiaceae, Acanthaceae, Apocynaceae, and Menispermaceae were the families with the highest CI values in the mentioned order. A high proportion of all the 3188 Karen use reports were used to treat digestive, general and unspecified, musculoskeletal, and skin disorders.

## 1. Introduction

Plants have been part of the human culture since pre-historical times. Humanity has accumulated knowledge of medicinal plants to combat diseases and bad health over innumerable generations. Although we currently develop scientific knowledge and cutting-edge technologies for treating disease and other health conditions, people in many parts of the world still depend on local knowledge and medicinal plants as the first or only option for treatments [1]. Moreover, traditional medicinal plant knowledge is invaluable for modern drug discovery, and many modern medicines were developed from such traditional knowledge [2,3,4]. Because of the importance of medicinal plants for both poor villagers and for industrial drug discovery, the rapid erosion of traditional knowledge is of global concern [5,6,7], and there is an urgent need for ethnobotanists to document and conserve this valuable knowledge before it is completely lost.

Thailand lies within one of the world’s biodiversity hotspots, which are areas with high levels of endemism and severe threats to their biota [8]. The country houses over 11,000 plant species [9], and is also home to a very large variety of ethnic minority populations [10,11]. Among these ethnic minorities, the Karen are the largest group among the so-called hill-tribe people of northern Thailand [10]. The Karen migrated from Myanmar to Thailand starting in the 18^th^ century because of wars [12]. Today, approximately 549,400 Karen live in 1930 villages in nine northern provinces and seven central and western provinces of Thailand [13].

The Karen have several characteristics that distinguish them from other hill-tribes in Thailand. The Karen language is in the Sino-Tibetan language family [10]. They wear distinct traditional dresses. Karen men wear red traditional shirts and the women wear two different types of dresses. Married women have tops with black or dark background colors that are decorated with colorful threads or dried millets. Girls and unmarried women wear white long dresses [14]. The Karen construct their villages near streams in hilly forests areas where they can farm the land. Traditionally, the Karen practice slash-and-burn agriculture, growing rice for their everyday consumption and for feeding their livestock [15]. Many Karen households prefer to cultivate useful plants in their home gardens [16]. More than 90% of the Karen in Thailand practice animism and the remainder have converted to become Buddhist or Christians. Their traditional culture and rituals are related to ancestral spirits, house spirits, forest spirits, farm spirits, land spirits, and so forth. [17]. For the treatment of sickness, the Karen depends on a large body of traditional knowledge about medicinal plants, especially herbs for the treatment of stomachaches, diarrhea, coughs, fevers, infectious diseases, and also plants for tonic and refreshment [14,18,19,20]. Therefore, the Karen hold a vast amount of medicinal plant knowledge.

The first study of Karen ethnobotany in Thailand appeared almost three decades ago [21]. After that, several graduate students reported on Karen ethnomedicinal uses in their theses based on studies in single villages, with a few exceptions of studies that included several villages [22,23]. Two recent studies of medicinal plants used in 12 Karen villages focused on Species Distribution Modelling (SDM) [24] and digestive system disorders [19]. Another ethnobotanical study of four Karen villages focused on medicinal legumes [25]. Our previous analysis of ethnomedicinal plant diversity in Thailand, based on references up until 2014, documented a huge number of species used by 19 different ethnic groups inhabiting 121 villages in Thailand. The Karen was the most extensively studied ethnic minority, representing 20 communities or one-sixth of the 121 studied villages [26,27]. The publications that have appeared since the beginning of Thai ethnobotanical studies until 2019 show that Karen is the most extensively studied group among ethnic minorities in Thailand [28]. Consequently, a very important part of the documented Thai ethnomedicinal knowledge is based on studies of the Karen. However, the number of Karen medicinal plants is still uncertain.

This study aims to present comprehensive information on Karen ethnomedicinal plant knowledge based on all the available reports originating from the beginning of the Thai ethnobotanical studies until the present. Based on these available reports, we included all data about medicinal plants used by Karen living in 31 villages distributed over eight provinces, namely Chiang Mai, Chiang Rai, Phetchaburi, Ratchaburi, Kanchanaburi, Mae Hong Son, Phrae, and Tak. Specifically, we aimed to answer the following questions: (1) How many plant species and families are used for medicine by the Karen in Thailand? (2) Which plant species and families were the most used by the Karen in Thailand? (3) Which medicinal health categories did the Karen most commonly treat with medicinal plants? (4) How well is ethnomedicinal knowledge distributed among the Karen?

## 2. Results and Discussion

### 2.1. Ethnomedicinal Use Reports

**Figure 1:**
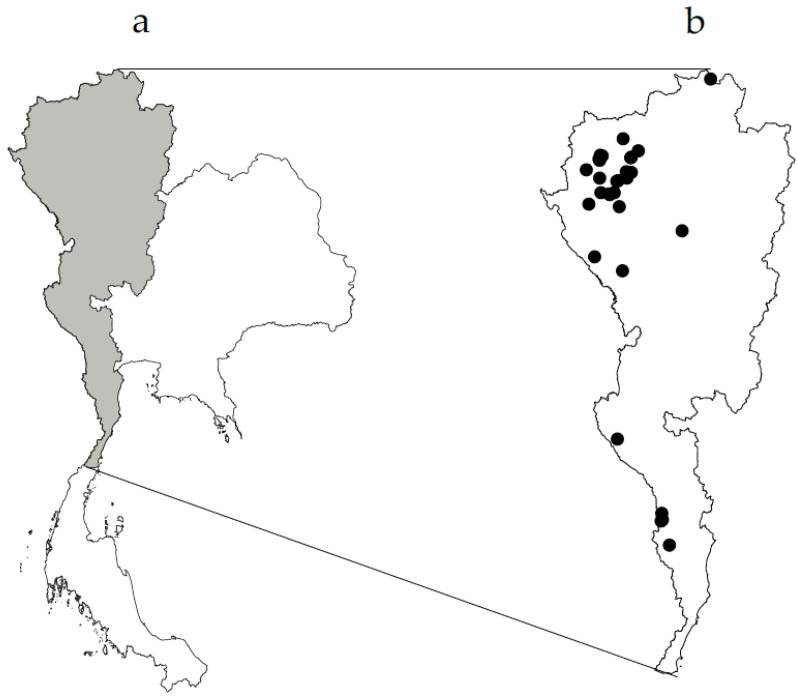
The distribution of Karen (grey shade) in Thailand (**a**) and the locations of 31 Karen villages from which data were obtained for this study (**b**).

The data from the 20 available references covering 31 Karen villages (Figure 1) included 3188 use reports for medicinal plants. These reports were based on the uses of 732 species in 150 plant families. The 31 villages with available data represent only 1.6% of the 1930 Karen villages in Thailand but still reveal the tremendous diversity of ethnomedicinal plants used. The 31 villages are located in eight provinces. Most of the studied villages are located in northern Thailand and some in western Thailand. Still, this is the most complete dataset on Karen ethnomedicinal plants. Even if the Karen population makes up less than 1% percent of the entire 70 million Thai population, the 732 medicinal plant species that they know account for more than one-third of all the ethnomedicinal species recorded in the country [27], and they represent 2.6% of the world’s recorded medicinal plants [29]. This shows that the Karen people guard vast amounts of ethnomedicinal knowledge. Because many modern medicines were developed from traditional knowledge [2,3,4], the ethnomedicinal knowledge of the Karen in Thailand might be useful for humanity as the source of essential knowledge for industrial drug development.

### 2.2. Cultural Importance Index (CI)

Eleven medicinal species had CI values of over one (1.00) (Supplementary Materials, Table S1). The top 10 species with the highest CI values together accounted for 389 use reports. These species were traditionally used for various treatments (Supplementary Materials, Table S2). *Chromolaena odorata* (L.) R.M.King and H.Rob had the highest CI value, and many of its use reports related to wound healing (Supplementary Materials, Table S2). This species was also the most used species among all the Thai ethnomedicinal plant species [27]. It has traditionally been used to stop bleeding, and the extracts of its leaves are known to promote fibroblast and epithelial cell growth [30], which makes sense relative to its use for wound healing. *Biancaea sappan* (L.) Tod. ranked second and was used to treat a number of ailments in the categories of digestive and musculoskeletal health conditions of the male and female reproductive organs, pregnancy, and related nutritional disorders (Supplementary Materials Table S2). The third most used Karen medicinal plant was *Tinospora crispa* (L.) Hook. f. and Thomson. This plant was also commonly mentioned in the study of all Thai ethnomedicinal plants [27]. The plant produces alkaloids, flavonoids, flavone glycosides, triterpenes, diterpenes, diterpene glycosides lactones, sterols, lignans, and nucleosides and it has various pharmacological effects, such as being anti-inflammatory, antioxidant, immunomodulatory, cytotoxic, antimalarial, cardioprotective, and anti-diabetic [31]. *Elephantopus scaber* L., *Sambucus javanica* Blume, *Scoparia dulcis* L., *Ricinus communis* L., *Zingiber ottensii* Valeton, *Thunbergia laurifolia* Lindl., *Blumea balsamifera* (L.) DC., and *Curcuma longa* L. also had high CI values and the records showed that these species were used in many categories (Supplementary Materials, Tables S1 and S2). The pattern was found when taking all the Karen villages together and also in some individual villages [32] and among other ethnic minorities in Thailand, such as the Hmong, the Khamu, the Lua, and the Mien [33]. When the same plant is used for the same purposes by different ethnicities, it suggests that it could have biologically based therapeutic effects and that it would be a good candidate for further pharmacological studies.

At the family level, Leguminosae had the highest CI values, indicating that a large proportion of the medicinal species used by the Karen would belong to this family (Supplementary Materials, Table S3). With 19,000 species, it is the third largest of all plant families [34] and also one of the largest plant families in the Thai flora, where it is represented by about 600 species [9]. On the global scale, Leguminosae have more medicinal species than any other families [29], and in Thailand, it has the highest Family Use Value of all ethnomedicinal plant families [27]. Many of the plant families with high CI values in this study of the Karen were also important providers of ethnomedicinal species among other ethnic minorities in Thailand in general [35].

### 2.3. Ethnomedicinal Use Categories

When studying medicinal plants, it important to be able to refer the information given about the diseases and health conditions treated to a common framework to have a meaningful discussion of the results. One well respected standard is the International Classification of Diseases (ICD), which now is updated to ICD-11 (www.who.int/classifications/icd/en/), and another is the International Classification of Primary Care (ICPC), updated to ICPC-2 (www.who.int/classifications/icd/adaptations/icpc2/en/). A third standard is the Economic Botany Data Collection Standard—EBDCS [36], which is widely used among ethnobotanists and which makes comparisons with other ethnobotanical studies easier. Each standard has its weaknesses and advantages [37]. Although EBDCS is popular among ethnobotanists, it has some limitations. Its categories have not been updated since the original publication was published 25 years ago, even if some additional use categories have been suggested, such as cultural diseases and disorders and ritual and magical uses [38]. The ICPC-2 has more detail than the EBDCS and also it is more suitable to classified diseases to compare with modern medical treatments.

**Figure 2:**
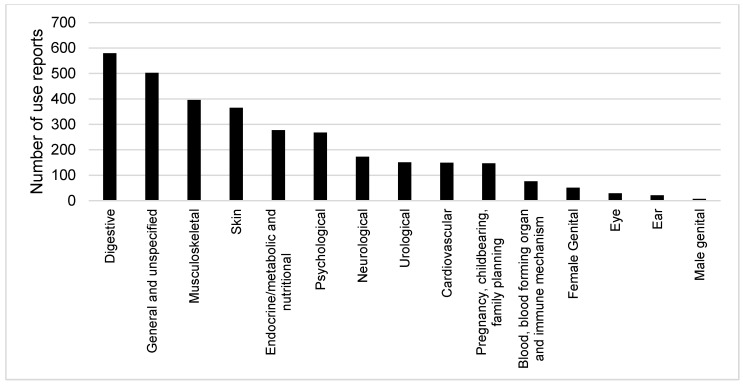
Numbers of use reports of Karen medicinal plants in 15 the International Classification of Primary Care (ICPC)-2 medicinal use categories.

The 3188 use reports of Thai Karen ethnomedicinal knowledge could be assigned to 15 categories in the ICPC-2. Digestive health conditions accounted for 18% of the overall number of use reports followed by the category of general and unspecified health conditions, which accounted for 16% of the use reports. The health categories in the third and fourth places were related to musculoskeletal and skin conditions, and together these four categories covered half of all the ethnomedicinal use reports. The number of use reports in each category of blood/blood forming organ and immune mechanism, female genital, eye, ear, and male genital health conditions accounted for less than 5% (Figure 2). The high number of uses relating to musculoskeletal conditions most likely relates to the Karen lifestyle, which involves much farming and rural livelihoods that may generate symptoms and illnesses related to this category, such as having muscle pain from hard physical labor. The many digestive system disorders involve diarrhea and intestinal worms, which are health conditions related to consuming uncooked or unclean food, having stomachache from unscheduled meals, and so forth.

### 2.4. Informant Consensus Relating to Karen Ethnomedicinal Uses

The four categories mentioned above as those with most use records also had high ICF (Informant Consensus Factor) values (Table 1), indicating that the medicinal plants used were much the same among the villages. Moreover, ICF values showed that Karen medicinal plants used for treatments related to digestive health, musculoskeletal system disorders, general and unspecified, endocrine/metabolic and nutritional, respiratory, and skin health conditions were not random (Table 1). However, the ICF values shown in this study were much lower than the possible maximum value (1.0) which can be calculated from the equation. This might be affected by many factors, such as the small number of studied villages or the independently developing traditional knowledge of villages in Thailand [26]. To include a higher number of studied villages would improve our understanding of this issue.

We suggest that Karen ethnomedicinal knowledge could be valuable for modern drug discoveries. In this context, it is worrying that much Karen traditional knowledge will disappear soon because of deforestation, urbanization, commercialization, and globalization. The issue of traditional knowledge erosion is of global concern [5,6,7]. Although this study collected the most substantial dataset on Thai Karen ethnomedicinal plants, it is imperative to visit more Karen villages to conserve Karen ethnomedicinal knowledge before it is forgotten. Our study shows that there have been comprehensive studies in northern Thailand, especially in the Chiang Mai province (Figure 1). However, many provinces with substantial Karen populations have not had their ethnomedicinal knowledge recorded yet [28]. It is, therefore, urgent to perform ethnomedicinal research in many additional Karen villages in Thailand. An ethnobotanical study in northwestern South America found that, although there were 255 publications in the regions published over the past 60 years, the studies could not cover all the ethnobotanical knowledge [39]. Our previous study [26] showed that different villages had different ethnomedicinal plant knowledge even when they were of the same ethnic group. Hence, we recommend that additional Karen villages should have their knowledge documented, as well as training for skills that are not able to be documented, to keep it for the next generations and also for the indirect benefits of other people as a source of drug discoveries. The highest concern is that there are many Karen villages in western Thailand [10] but that only a few Karen villages (Figure 1) and other ethnic minorities [28] have been ethnobotanically studied. This area is close to the Bangkok metropolis, and urbanization will accelerate the erosion of traditional knowledge in this area. Therefore, we suggest that Karen villages in this area should be prioritized for research to document their traditional knowledge as soon as possible.

## 3. Materials and Methods

### 3.1. Data Collection

This study used previously assembled data on medicinal plants used by the Karen. The data were collected from university libraries, online databases (Scopus, PubMed, and Google Scholar), and Thai journals. We also searched the website of the Thai Library Integrated System (www.tdc.thailis.or.th), which houses most postgraduate student theses and scientific reports from all higher educational institutes in Thailand. We merge the duplicated data, for example, when published journal articles were based on data from a thesis, the duplicated records from other sources were excluded. Ethnomedicinal data that did not specify the scientific names of plants used were also excluded, together with data from studies that had not used proper scientific procedures for gathering the data. All in all, our data were derived from one book, one scientific report, three journal articles, and 15 theses (Supplementary Materials Table S1). In total, we found data from 31 Karen villages in Thailand (Figure 1). All the scientific species and family names were updated according to The World Checklist of Vascular Plants (WCVP) (wcvp.science.kew.org). This study prefers to use the name Leguminosae instead of Fabaceae.

Finally, each use report was classified into a medicinal category of the International Classification of Primary Care (ICPC) updated to ICPC-2 (www.who.int/classifications/icd/adaptations/icpc2/en/).

### 3.2. Important Plant Taxa

The relative importance of the Karen medicinal plant species and families were calculated using the Cultural Importance index (CI) [40] as:

CI = UR/N,(1)

where UR is the total number of use reports for a species and N is the total number of informants in the interview. Because the data in this study are meta-data derived from previous reports that did not connect each use report to a single informant, we counted each studied village as a “pseudoinformant” [27] to replace an individual person “informant” who was a person giving ethnomedicinal data. The higher the CI value, the higher the proportion of informants that knew the species, or in our case, the higher the CI value, the more villages used the species. On the other hand, a CI close to zero (0) implies that a plant and its ethnomedicinal uses were known in only one or a few of the sampled villages.

To see how uniform the ethnomedicinal plant knowledge was between the studied villages, we calculated the Informant Consensus Factor, which was modified from [41] as:

ICF = (Nur − Nt)/(Nur − 1),(2)

where Nur is the number of use reports in a category and Nt is the number of species or taxa used for treatment in that category. The value of ICF is close to or equals one when the same species is used for the same purposes in most or all of the studied villages. On the other hand, ICF values close to zero mean that the plants were used randomly and not in a systematic and shared way between the villages.

## 4. Conclusions

The Thai Karen guard a vast knowledge of a high diversity of ethnomedicinal plants that they use for treatments of a very broad spectrum of health conditions such as digestive, musculoskeletal, general and unspecified, skin, endocrine/metabolic and nutritional, and genital disorders. Although the Karen account for less than one percent of Thailand’s population, they use more than one third of the medicinal plant species known in the country. Many species, such as *Biancaea sappan*, *Chromolaena odorata*, and *Tinospora crispa*, as well as several plant families—e.g., Leguminosae, Asteraceae, Zingiberaceae, Euphorbiaceae, Lamiaceae, and others—were important medicinal taxa for the Karen in Thailand. Even though this study gathered the most complete set of information on the ethnomedicinal plants used by Karen in Thailand, we suggest that more ethnomedicinal plant data should be collected from other Karen villages. That would give us a more robust base for future research and the protection of traditional knowledge.

## Figures and Tables

**Table 1:** Informant Consensus Factors (ICF) for medicinal plant use ICPC-2 disorder categories among the Karen in Thailand.

Disorder Category	ICF
Ear	0.50
Musculoskeletal	0.47
Skin	0.46
Psychological	0.45
Blood, blood forming organ and immune mechanism	0.44
General and unspecified	0.44
Digestive	0.42
Urological	0.40
Pregnancy, childbearing, family planning	0.38
Endocrine/metabolic and nutritional	0.35
Eye	0.29
Neurological	0.29
Female Genital	0.28
Cardiovascular	0.16
Male genital	0.00

## Supplementary Materials

The following are available online at https://www.mdpi.com/2223-7747/9/7/813/s1, Table S1: CI values of medicinal plants used by Karen in Thailand, Table S2: Number of use reports for each symptom/treatment/uses of the top ten most used plant species by Karen in Thailand, Table S3: CI values of medicinal plants families used by Karen in Thailand.
