# Supplementary material for: Ethnomedicinal Plant Knowledge of the Karen in Thailand
Source: Plants (Basel). 2020 Jun 29;9(7):813. doi: 10.3390/plants9070813 (PMC7412177; doi:10.3390/plants9070813)
Supplement: Supplementary file 1 [file plants-09-00813-s001.pdf]

Supplementary Materials

Ethnomedicinal plant knowledge of the Karen in Thailand

Methee Phumthum, Rapeeporn Kantasrila, Sukhumaabhorn Kaewsangsai, Henrik Balslev, Angkhana Inta

List of data sources

1. Anderson, E.F., 1993. Plants and People of the Golden Triangle Ethnobotany of the Hill Tribes of Northern Thailand. Timber Press, Inc., Southwest Portland.
2. Junkhonkaen, J., 2012. ethnobotany of Ban Bowee, Amphoe Suan Phueng, Changwat Ratchaburi. Master thesis, Kasetsart University, Kasetsart University Library, Bangkok.
3. Junsongduang, A., 2014. Roles and importance of sacred Forest in biodiversity conservation in Mae Chaem District, Chiang Mai Province. PhD thesis, Biology, Chiang Mai University Library, Chiang Mai.
4. Kaewsangsai S. Ethnobotany of Karen in Khun Tuen Noi Village, Mae Tuen Sub-district, Omkoi District, Chiang Mai Province. Master thesis. Chiang Mai: Chiang Mai University; 2017.
5. Kamwong, K., 2010. Ethnobotany of Karens at Ban Mai Sawan and Ban Huay Pu Ling, Ban Luang Sub-District, Chom Thong District, Chiang Mai Province, Master thesis, Biology. Chiang Mai University Library, Chiang Mai.
6. Kantasrila, R. Ethnobotany fo Karen at Ban Wa Do Kro, Mae Song Sub-district, Tha Song Yang District, Tak Province. Master thesis. Chiang Mai: Chiang Mai University Library. 2016.
7. Klibai, A. 2013. Self-care with indigenous medicine of long-eared Karen ethnic group: Case study Ban Mae Sin, Ban Kang Pinjai, Ban Slok, Wang Chin district, Phrae province. Master thesis, Surin Rajabhat University.
8. Mahawongsanan, A., 2008. Change of herbal plants utilization of the Pgn K'nyau : A case study of Ban Huay Som Poy, Mae Tia Watershed, Chom Thong District, Chiang Mai Province. Master thesis, Biology. Chiang Mai University, Chiang Mai University Library.
9. Moonjai, J. Ethnobotany of Ethnic Group in Mae La Noi District, Mae Hong Son Province. Master's Thesis, Chiang Mai University, Chiang Mai, Thailand, 2017.
10. Prachuabaree, L., 2008. Medicinal plants of Karang hill tribe in Baan Pong-lueg, Kaeng Krachan District, Phetchaburi Province. Master thesis, Pharmacy. Silpakorn University, Nakhonpathom.
11. Pongamornkul, W. 2003. An ethnobotanical study of the Karen at Ban Yang Pu Toh and Ban Yang Thung Pong, Chiang Dao district, Chiang Mai province. Bachelor degree thesis, Biology. Chiang Mai University Library, Chiang Mai.
12. Puling, W. 2001. Ethnobotany of Karen for studying medicinal plants at Angka Noi and Mae Klang villages, Chomthong district, Chiang Mai. Bachelor degree thesis, Biology. Chiang Mai University Library, Chiang Mai.
13. Sonsupub, B., 2010. Ethnobotany of karen community in Raipa village, Huaykhayeng subdistrict, Thongphahpume district, Kanchanaburi province. Master thesis, Hoticulture. Kasetsart University, Bangkok.
14. Sukkho, T., 2008. A survey of medicinal plants used by Karen people at Ban Chan and Chaem Luang Subdidtricts, Mae Chaem district, Chiang Mai province. Biology. Chiang Mai University Library, Chiang Mai.
15. Sutjaritjai, N., Wangpakapattanawong, P., Balslev, H. & Inta, A. 2019. Sutjaritjai, N., P. Wangpakapattanawong, H. Balslev and A. Inta (2019). "Traditional Uses of Leguminosae among the Karen in Thailand. Plants 8(12). https://doi.org/10.3390/plants8120600.

16. Tangjitman, K. Vulnerability prediction of medicinal plants used by Karen people in Chiang Mai province to climatic change using species distribution model (SDM). Doctor of Philosophy, Chiang Mai University, Chiang Mai, Thailand, 2014.
17. Tangjitman, K. Ethnobotany of the Karen at Huay Nam and Nong Ta Dang Villages, TanaosriSubdistrict, SuanphuangDistrict, Ratchaburi Province. Muban Chombueng Rajabhat University. 2016.
18. Trisonthi, S., Trisonthi, P. 1995. Ethnobotany of Karen in Mae Hae Nua village, Mae Na Jorn subdistrict, Mae Chaem district, Chiang Mai. Scientific report. CU Library, Bangkok.
19. Trisonthi, C.; Trisonthi, P. Ethnobotanical study in Thailand, a case study in Khun Yuam district Maehongson province. *Thai J Bot* **2009**, *1*, 1-23.
20. Winijchaiyanan, P., 1995. Ethnobotany of Karen in Chiang Mai. Master thesis, Biology. Chiang Mai University Library, Chiang Mai.

Table S1 CI values of medicinal plants used by Karen in Thailand

Species	CI
Abrus precatorius L.	0.03
Acacia catechu (L.f.) Willd.	0.03
Acacia concinna (Willd.) DC.	0.39
Acalypha kerrii Craib	0.03
Acalypha siamensis Oliv. ex Gage	0.03
Acalypha spiciflora Burm.f.	0.06
Acanthus montanus (Nees) T.Anderson	0.06
Achyranthes aspera L.	0.16
Acmella oleracea (L.) R.K.Jansen	0.23
Acmella paniculata (Wall. ex DC.) R.K.Jansen	0.06
Acorus calamus L.	0.77
Acrocarpus fraxinifolius Wight & Arn.	0.06
Actinodaphne henryi Gamble	0.03
Actinoscirpus grossus (L.f.) Goetgh. & D.A.Simpson	0.10
Adenanthera pavonina L.	0.06
Adenia viridiflora Craib	0.03
Aeginetia indica L.	0.10
Aegle marmelos (L.) Corrêa	0.03
Aeschynomene americana L.	0.06
Aesculus assamica Griff.	0.35
Afzelia xylocarpa (Kurz) Craib	0.03
Agapetes hosseana Diels	0.06
Agave americana L.	0.03
Ageratina adenophora (Spreng.) R.M.King & H.Rob.	0.48
Ageratum conyzoides L.	0.29
Aglaia elliptica (C.DC.) Blume	0.03
Aglaia lawii (Wight) C.J.Saldanha	0.06
Albizia chinensis (Osbeck) Merr.	0.03
Albizia procera (Roxb.) Benth.	0.03

Alisma plantago-aquatica L.	0.03
Allamanda cathartica L.	0.03
Allium ascalonicum L.	0.06
Allium sativum L.	0.13
Aloe vera (L.) Burm.f.	0.32
Alpinia galanga (L.) Willd.	0.16
Alpinia malaccensis (Burm.f.) Roscoe	0.39
Alpinia roxburghii Sweet	0.03
Alpinia zerumbet (Pers.) B.L.Burtt & R.M.Sm.	0.13
Alstonia rostrata C.E.C.Fisch.	0.23
Alstonia scholaris (L.) R.Br.	0.52
Alternanthera sessilis (L.) R.Br. ex DC.	0.03
Amalocalyx microlobus Pierre ex Spire	0.10
Amaranthus blitum L.	0.06
Amaranthus spinosus L.	0.06
Amphineurion marginatum (Roxb.) D.J.Middleton	0.13
Anacardium occidentale L.	0.06
Anacolosa ilicoides Mast.	0.03
Ananas comosus (L.) Merr.	0.03
Anaxagorea luzonensis A.Gray	0.03
Andrographis paniculata (Burm.f.) Nees	0.16
Angiopteris evecta (G.Forst.) Hoffm.	0.32
Annona squamosa L.	0.10
Anogeissus acuminata (Roxb. ex DC.) Wall. ex Guill. & Perr.	0.10
Anredera cordifolia (Ten.) Steenis	0.16
Antidesma acidum Retz.	0.10
Antidesma bunius (L.) Spreng.	0.03
Antidesma ghaesembilla Gaertn.	0.13
Antidesma sootepense Craib	0.06
Aphaenandra uniflora (Wall. ex G.Don) Bremek.	0.03
Aphananthe aspera (Thunb.) Planch.	0.03
Aporosa villosa (Lindl.) Baill.	0.06
Aquilaria crassna Pierre ex Lecomte	0.06
Aralia decaisneana Hance	0.03
Archidendron clypearia (Jack) I.C.Nielsen	0.35
Ardisia polycephala Wall. ex A.DC.	0.06
Areca catechu L.	0.13
Arisaema auriculatum Buchet	0.03
Aristolochia tagala Cham.	0.13
Artemisia atrovirens Hand.-Mazz.	0.19
Artemisia austroyunnanensis Ling & Y.R.Ling	0.06
Artocarpus gomezianus Wall. ex Trécul	0.03
Artocarpus heterophyllus Lam.	0.10

Artocarpus hypargyreus Hance ex Benth.	0.03
Artocarpus lacucha Buch.-Ham.	0.06
Asparagus filicinus Buch.-Ham. ex D.Don	0.39
Asparagus racemosus Willd.	0.06
Aspidistra elatior Blume	0.23
Asplenium nidus L.	0.03
Averrhoa carambola L.	0.13
Ayenia andamensis (Kurz) Christenh. & Byng	0.03
Azadirachta indica A.Juss.	0.10
Baccaurea ramiflora Lour.	0.03
Baliospermum calycinum Müll.Arg.	0.10
Baliospermum solanifolium (Burm.) Suresh	0.29
Bambusa bambos (L.) Voss	0.06
Barleria lupulina Lindl.	0.06
Barleria strigosa Willd.	0.06
Barringtonia macrostachya (Jack) Kurz	0.16
Basella alba L.	0.03
Bauhinia nervosa (Benth.) Baker	0.03
Bauhinia ornata Kurz	0.06
Bauhinia pulla Craib	0.03
Benincasa hispida (Thunb.) Cogn.	0.03
Berberis napaulensis (DC.) Spreng.	0.03
Berchemia floribunda (Wall.) Brongn.	0.10
Betula alnoides Buch.-Ham. ex D.Don	0.68
Biancaea sappan (L.) Tod.	1.58
Bidens bipinnata L.	0.03
Bidens pilosa L.	0.29
Biophytum umbraculum Welw.	0.03
Bixa orellana L.	0.03
Blainvillea acmella (L.) Philipson	0.03
Blumea balsamifera (L.) DC.	0.84
Blumea fistulosa (Roxb.) Kurz	0.03
Blumea lacera (Burm.f.) DC.	0.06
Boehmeria glomerulifera Miq.	0.03
Boesenbergia rotunda (L.) Mansf.	0.13
Brachypterum scandens (Roxb.) Miq.	0.03
Breynia androgyna (L.) Chakrab. & N.P.Balakr.	0.13
Breynia quadrangularis (Willd.) Chakrab. & N.P.Balakr.	0.06
Breynia retusa (Dennst.) Alston	0.10
Breynia vitis-idaea (Burm.f.) C.E.C.Fisch.	0.03
Bridelia glauca Blume	0.03
Bridelia ovata Decne.	0.03
Broussonetia papyrifera (L.) L'Hér. ex Vent.	0.03

Brucea javanica (L.) Merr.	0.13
Brucea mollis Wall. ex Kurz	0.03
Bryophyllum pinnatum (Lam.) Oken	0.16
Buchanania cochinchinensis (Lour.) M.R.Almeida	0.03
Buddleja asiatica Lour.	0.39
Buxus cochinchinensis Pierre ex Gagnep.	0.03
Cajanus cajan (L.) Huth	0.16
Calanthe cardioglossa Schltr.	0.10
Callicarpa arborea Roxb.	0.10
Callicarpa rubella Lindl.	0.13
Calophyllum polyanthum Wall. ex Choisy	0.03
Calotropis gigantea (L.) W.T.Aiton	0.06
Camellia sinensis (L.) Kuntze	0.13
Cananga odorata (Lam.) Hook. f. & Thomson	0.06
Canna indica L.	0.23
Canscora andrographioides Griff. ex C.B.Clarke	0.03
Capsicum annuum L.	0.03
Capsicum frutescens L.	0.03
Cardiospermum halicacabum L.	0.06
Careya arborea Roxb.	0.16
Carica papaya L.	0.10
Cascabela thevetia (L.) Lippold	0.03
Cassia fistula L.	0.29
Cassytha filiformis L.	0.61
Catharanthus roseus (L.) G.Don	0.03
Causonis trifolia (L.) Mabb. & J.Wen	0.03
Cayratia pedata Gagnep.	0.19
Celastrus paniculatus Willd.	0.16
Celosia argentea L.	0.03
Celtis tetrandra Roxb.	0.26
Celtis timorensis Span.	0.16
Centella asiatica (L.) Urb.	0.71
Centipeda minima (L.) A.Braun & Asch.	0.03
Chionanthus ramiflorus Roxb.	0.03
Chisocheton cumingianus (C.DC.) Harms	0.03
Chloranthus elatior Link	0.68
Chromolaena odorata (L.) R.M.King & H.Rob.	1.61
Chrozophora tinctoria (L.) A.Juss.	0.48
Cinnamomum camphora (L.) J.Presl	0.13
Cinnamomum iners (Reinw. ex Nees & T.Nees) Blume	0.03
Cinnamomum subavenium Miq.	0.19
Cissampelos hispida Forman	0.13
Cissus bicolor Domin	0.13

Cissus discolor Blume	0.16
Cissus hastata Miq.	0.13
Cissus quadrangularis L.	0.06
Citrus × aurantiifolia (Christm.) Swingle	0.23
Citrus × reticulata Blanco	0.03
Citrus cavaleriei H.Lév. ex Cavalerie	0.06
Citrus hystrix DC.	0.10
Citrus maxima (Burm.) Merr.	0.03
Citrus medica L.	0.06
Clausena anisata (Willd.) Hook. f. ex Benth.	0.06
Clausena excavata Burm.f.	0.23
Clausena harmandiana (Pierre) Guillaumin	0.03
Clausena lenis Drake	0.03
Cleidion javanicum Blume	0.39
Clematis smilacifolia Wall.	0.16
Clerodendrum chinense (Osbeck) Mabb.	0.06
Clerodendrum infortunatum L.	0.03
Clerodendrum japonicum (Thunb.) Sweet	0.03
Clerodendrum nutans Wall. ex Jack	0.10
Clerodendrum paniculatum L.	0.23
Clerodendrum petasites (Lour.) S.Moore	0.13
Clinacanthus nutans (Burm.f.) Lindau	0.03
Cnestis palala (Lour.) Merr.	0.10
Coccinia grandis (L.) Voigt	0.03
Cocculus laurifolius DC.	0.03
Cocos nucifera L.	0.06
Codariocalyx motorius (Houtt.) H.Ohashi	0.10
Codiaeum variegatum (L.) Rumph. ex A.Juss.	0.03
Coffea canephora Pierre ex A.Froehner	0.03
Coix lacryma-jobi L.	0.58
Coleus amboinicus Lour.	0.26
Colocasia esculenta (L.) Schott	0.03
Colquhounia elegans Wall. ex Benth.	0.13
Combretum deciduum Collett & Hemsl.	0.03
Combretum indicum (L.) DeFilipps	0.13
Combretum procursum Craib	0.03
*Combretum pun*ctatum Blume	0.03
Commelina benghalensis L.	0.03
Commelina diffusa Burm.f.	0.03
Congea tomentosa Roxb.	0.03
Cordyline fruticosa (L.) A.Chev.	0.06
*Crassocephalum crepidi*oides (Benth.) S.Moore	0.03
Crateva adansonii DC.	0.03

Crateva religiosa G.Forst.	0.16
Cratoxylum formosum (Jack) Benth. & Hook. f. ex Dyer	0.48
Crinum asiaticum L.	0.13
Crotalaria alata Buch.-Ham. ex D.Don	0.06
Crotalaria albida B.Heyne ex Roth	0.03
Crotalaria assamica Benth.	0.03
Crotalaria breviflora DC.	0.13
Crotalaria pallida Aiton	0.03
Crotalaria sessiliflora L.	0.03
Crotalaria verrucosa L.	0.06
Croton acutifolius Esser	0.03
Croton kongensis Gagnep.	0.10
Croton persimilis Müll.Arg.	0.23
Croton robustus Kurz	0.06
Croton sepalinus Airy Shaw	0.06
Croton stellatopilosus H.Ohba	0.03
Curculigo capitulata (Lour.) Kuntze	0.10
Curculigo latifolia Dryand. ex W.T.Aiton	0.19
Curcuma aeruginosa Roxb.	0.23
Curcuma elata Roxb.	0.06
Curcuma longa L.	0.94
Curcuma mangga Valeton & Zijp	0.03
Curcuma sessilis Gage	0.03
Curcuma zedoaria (Christm.) Roscoe	0.23
Cuscuta chinensis Lam.	0.26
Cyanthillium cinereum (L.) H.Rob.	0.06
Cycas siamensis Miq.	0.03
Cyclea barbata Miers	0.55
Cyclocodon lancifolius (Roxb.) Kurz	0.16
Cymbidium bicolor Lindl.	0.10
Cymbidium haematodes Lindl.	0.03
Cymbopogon citratus (DC.) Stapf	0.23
Cymbopogon nardus (L.) Rendle	0.10
Cyperus rotundus L.	0.03
Dactylicapnos scandens (D.Don) Hutch.	0.13
Dactyloctenium aegyptium (L.) Willd.	0.03
Dalbergia cana Graham ex Kurz	0.03
Dalbergia cultrata T.S.Ralph	0.06
Dalbergia foliacea Wall. ex Benth.	0.03
Dalbergia ovata Graham ex Benth.	0.10
Dalbergia retusa Hemsl.	0.03
Dalbergia stipulacea Roxb.	0.19
Dalbergia velutina Benth.	0.06

Dalrympelea pomifera Roxb.	0.16
Datura metel L.	0.06
Dendrocalamus brandisii (Munro) Kurz	0.10
Dendrocalamus hamiltonii Nees & Arn. ex Munro	0.13
Dendrocalamus strictus (Roxb.) Nees	0.10
Dendrophthoe pentandra (L.) Miq.	0.16
Derris elliptica (Wall.) Benth.	0.26
Derris montana Benth.	0.03
Desmodium oblongum Wall. ex Benth.	0.19
Desmodium velutinum (Willd.) DC.	0.06
Desmos dumosus (Roxb.) Saff.	0.06
Desmos macrocarpus Bân	0.13
Dianella ensifolia (L.) Redouté	0.03
Dienia ophrydis (J.Koenig) Seidenf.	0.03
Dillenia indica L.	0.03
Dillenia parviflora Griff.	0.03
Dillenia pentagyna Roxb.	0.03
Dimetia ampliflora (Hance) Neupane & N.Wikstr.	0.16
Dioscorea hispida Dennst.	0.16
Diospyros areolata King & Gamble	0.03
Diospyros castanea (Craib) H.R.Fletcher	0.06
Diospyros glandulosa Lace	0.03
Diospyros kaki L.f.	0.06
Diospyros mollis Griff.	0.03
Diospyros rhodocalyx Kurz	0.03
Diplazium esculentum (Retz.) Sw.	0.06
Dipterocarpus obtusifolius Teijsm. ex Miq.	0.06
Dischidia imbricata (Blume) Steud.	0.03
Dischidia major (Vahl) Merr.	0.03
Dischidia nummularia R.Br.	0.35
Disporum calcaratum D.Don	0.03
Dolichandrone serrulata (Wall. ex DC.) Seem.	0.03
Dracaena angustifolia (Medik.) Roxb.	0.03
Dracaena cochinchinensis (Lour.) S.C.Chen	0.03
Dracaena conferta Ridl.	0.03
Dracaena fragrans (L.) Ker Gawl.	0.06
Dracaena terniflora Roxb.	0.10
Dracontomelon dao (Blanco) Merr. & Rolfe	0.03
Drymaria cordata (L.) Willd. ex Schult.	0.10
Drynaria quercifolia (L.) J.Sm.	0.13
Duabanga grandiflora (Roxb. ex DC.) Walp.	0.16
Duhaldea cappa (Buch.-Ham. ex D.Don) Pruski & Anderb.	0.71
Dunbaria bella Prain	0.03

Dysoxylum excelsum Blume	0.03
Dysoxylum grande Hiern	0.26
Elaeagnus latifolia L.	0.03
Elephantopus scaber L.	1.26
Eleusine indica (L.) Gaertn.	0.06
Eleutherine bulbosa (Mill.) Urb.	0.45
Eleutherococcus trifoliatus (L.) S.Y.Hu	0.03
Elytranthe albida (Blume) Blume	0.03
Embelia ribes Burm.f.	0.13
Embelia sessiliflora Kurz	0.29
Embelia tsjeriam-cottam (Roem. & Schult.) A.DC.	0.03
Engelhardia spicata Lechen ex Blume	0.13
Ensete glaucum (Roxb.) Cheesman	0.16
Entada glandulosa Pierre ex Gagnep.	0.13
Entada rheedei Spreng.	0.13
Equisetum debile Roxb. ex Vaucher	0.13
Eriosema chinense Vogel	0.03
Eryngium foetidum L.	0.10
Erythrina stricta Roxb.	0.06
Erythrina subumbrans (Hassk.) Merr.	0.23
Etlingera elatior (Jack) R.M.Sm.	0.03
Eupatorium fortunei Turcz.	0.06
Euphorbia heterophylla L.	0.19
Euphorbia hirta L.	0.23
Euphorbia tirucalli L.	0.03
Eurycoma longifolia Jack	0.16
Exacum pteranthum Wall. ex G.Don	0.03
Falconeria insignis Royle	0.13
Fernandoa adenophylla (Wall. ex G.Don) Steenis	0.06
Ficus auriculata Lour.	0.26
Ficus capillipes Gagnep.	0.03
Ficus fistulosa Reinw. ex Blume	0.03
Ficus heterophylla L.f.	0.03
Ficus hispida L.f.	0.19
Ficus racemosa L.	0.13
Ficus sarmentosa Buch.-Ham. ex Sm.	0.03
Ficus semicordata Buch.-Ham. ex Sm.	0.03
Ficus virens Aiton	0.03
Flacourtia indica (Burm.f.) Merr.	0.26
Flacourtia jangomas (Lour.) Raeusch.	0.13
Flacourtia rukam Zoll. & Moritzi	0.03
Flemingia ferruginea Wall. ex Benth.	0.03
Flemingia lineata (L.) Aiton	0.16

Flemingia macrophylla (Willd.) Kuntze ex Merr.	0.16
Flemingia paniculata Wall. ex Benth.	0.03
Flemingia semialata Roxb. ex W.T.Aiton	0.06
Flemingia stricta Roxb.	0.10
Flemingia strobilifera (L.) W.T.Aiton	0.16
Flueggea leucopyrus Willd.	0.26
Flueggea virosa (Roxb. ex Willd.) Royle	0.03
Fraxinus griffithii C.B.Clarke	0.06
Garcinia xanthochymus Hook. f. *ex T.Anderson*	0.03
Gardenia jasminoides J.Ellis	0.03
Garuga pinnata Roxb.	0.03
Getonia floribunda Roxb.	0.03
Gigantochloa albociliata (Munro) Kurz	0.06
Glinus herniarioides (Gagnep.) Tardieu	0.03
Gluta usitata (Wall.) Ding Hou	0.03
Glycine max (L.) Merr.	0.03
Glycosmis pentaphylla (Retz.) DC.	0.03
Gmelina arborea Roxb. ex Sm.	0.81
Gnetum montanum Markgr.	0.10
Gomphrena celosioides Mart.	0.03
Gomphrena globosa L.	0.06
Gonocaryum lobbianum (Miers) Kurz	0.06
Grammatophyllum speciosum Blume	0.16
Gynostemma pentaphyllum (Thunb.) Makino	0.03
Gynura bicolor (Roxb. ex Willd.) DC.	0.03
Harrisonia perforata (Blanco) Merr.	0.23
Hedychium coronarium J.Koenig	0.06
Hedychium flavum Roxb.	0.03
Hedyotis pruinosa Wight & Arn.	0.03
Helicia nilagirica Bedd.	0.13
Heliciopsis terminalis (Kurz) Sleumer	0.16
Helicteres elongata Wall. ex Mast.	0.29
Helicteres hirsuta Lour.	0.03
Heliotropium indicum L.	0.10
Hellenia speciosa (J.Koenig) S.R.Dutta	0.58
Hemigraphis glaucescens (Nees) C.B.Clarke	0.03
Henslowia collettii Gamble	0.10
Henslowia sessilis Craib	0.13
Heteropanax fragrans (Roxb.) Seem.	0.06
Hibiscus rosa-sinensis L.	0.03
Hibiscus sabdariffa L.	0.03
Hippochaete debilis (Roxb. ex Vaucher) Holub	0.42
Hiptage benghalensis (L.) Kurz	0.29

Hiptage candicans Hook.f.	0.06
Holarrhena pubescens Wall. ex G.Don	0.10
Homonoia riparia Lour.	0.03
Hopea helferi (Dyer) Brandis	0.03
Hopea odorata Roxb.	0.03
Horsfieldia amygdalina (Wall.) Warb.	0.03
Houttuynia cordata Thunb.	0.23
Huangtcia renifolia (L.) H.Ohashi & K.Ohashi	0.03
Hultholia mimosoides (Lam.) Gagnon & G.P.Lewis	0.03
Hydnocarpus ilicifolius King	0.03
Hydrocotyle javanica Thunb.	0.23
Hydrocotyle umbellata L.	0.03
Hymenasplenium apogamum (N.Murak. & Hatan.) Nakaike	0.03
Hymenasplenium obscurum (Blume) Tagawa	0.03
Hymenodictyon orixense (Roxb.) Mabb.	0.06
Hypoestes phyllostachya Baker	0.10
Ilex umbellulata (Wall.) Loes.	0.03
Illigera trifoliata (Griff.) Dunn	0.19
Impatiens muscicola Craib	0.06
Imperata cylindrica (L.) P.Beauv.	0.39
Indigofera caloneura Kurz	0.13
Indigofera hendecaphylla Jacq.	0.06
Indigofera tinctoria L.	0.03
Ipomoea aquatica Forssk.	0.03
Ipomoea batatas (L.) Lam.	0.06
Ipomoea quamoclit L.	0.03
Iris domestica (L.) Goldblatt & Mabb.	0.03
Ixora cibdela Craib	0.03
Ixora henryi H.Lév.	0.13
Ixora lobbii Loudon ex King & Gamble	0.03
Jasminum decussatum Wall. ex G.Don	0.10
Jasminum laurifolium Roxb. ex Hornem.	0.10
Jasminum simplicifolium G.Forst.	0.03
Jatropha curcas L.	0.35
Jatropha podagrica Hook.	0.13
Justicia adhatoda L.	0.03
Justicia fragilis Dennst.	0.06
Justicia gendarussa Burm.f.	0.29
Kaempferia galanga L.	0.10
Kaempferia parviflora Wall. ex Baker	0.32
Kaempferia rotunda L.	0.10
Kopsia arborea Blume	0.13
Lablab purpureus (L.) Sweet	0.10

Lagerstroemia calyculata Kurz	0.10
Lagerstroemia floribunda Jack	0.03
Lagerstroemia speciosa (L.) Pers.	0.03
Lannea coromandelica (Houtt.) Merr.	0.03
Lantana camara L.	0.10
Lasia spinosa (L.) Thwaites	0.03
Leea indica (Burm.f.) Merr.	0.29
Leonotis nepetifolia (L.) R.Br.	0.03
Lepisanthes senegalensis (Poir.) Leenh.	0.03
Leucaena leucocephala (Lam.) de Wit	0.19
Lilium primulinum Baker	0.06
Limnophila aromatica (Lam.) Merr.	0.10
Litsea cubeba (Lour.) Pers.	0.81
Lophopetalum wallichii Kurz	0.03
Ludwigia hyssopifolia (G.Don) Exell	0.03
Luffa cylindrica (L.) M.Roem.	0.03
Lycopodiella cernua (L.) Pic.Serm.	0.06
Lygodium flexuosum (L.) Sw.	0.45
Macaranga denticulata (Blume) Müll.Arg.	0.03
Maclura cochinchinensis (Lour.) Corner	0.03
Maesa glomerata K.Larsen & C.M.Hu	0.06
Maesa junghuhniana Scheff.	0.06
Maesa ramentacea (Roxb.) A.DC.	0.03
Magnolia garrettii (Craib) V.S.Kumar	0.03
Mallotus philippensis (Lam.) Müll.Arg.	0.16
Mangifera indica L.	0.06
Mangifera quadrifida Jack	0.06
Mansoa alliacea (Lam.) A.H.Gentry	0.03
Maranta arundinacea L.	0.10
Markhamia stipulata (Wall.) Seem.	0.26
Martynia annua L.	0.06
Mayodendron igneum (Kurz) Kurz	0.06
Melastoma malabathricum L.	0.26
Melastoma sanguineum Sims	0.13
Melia azedarach L.	0.06
Melicope glomerata (Craib) T.G.Hartley	0.48
Melicope pteleifolia (Champ. ex Benth.) T.G.Hartley	0.23
Meliosma pinnata (Roxb.) Maxim.	0.03
Memecylon pauciflorum Blume	0.10
Mentha × villosa Huds.	0.16
Mesosphaerum suaveolens (L.) Kuntze	0.03
Microcos paniculata L.	0.19
Microcos sinuata (Wall. ex Mast.) Burret	0.03

Microcos tomentosa Sm.	0.06
Microglossa pyrifolia (Lam.) Kuntze	0.06
Microlepia matthewii Christ	0.06
Micromelum integerrimum (Roxb. ex DC.) Wight & Arn. ex M.Roem.	0.06
Micromelum minutum (G.Forst.) Wight & Arn.	0.13
Microtoena insuavis (Hance) Prain ex Briq.	0.06
Mikania cordata (Burm.f.) B.L.Rob.	0.03
Miliusa thorelii Finet & Gagnep.	0.77
Miliusa velutina (DC.) Hook.f. & Thomson	0.10
Millettia brandisiana Kurz	0.03
Millettia caerulea Baker	0.06
Millettia extensa (Benth.) Benth. ex Baker	0.03
Millettia pachycarpa Benth.	0.06
Millingtonia hortensis L.f.	0.29
Mimosa diplotricha C.Wright	0.06
Mimosa pigra L.	0.97
Mirabilis jalapa L.	0.03
Mitragyna hirsuta Havil.	0.03
Mitragyna rotundifolia (Roxb.) Kuntze	0.06
Momordica charantia L.	0.23
Momordica cochinchinensis (Lour.) Spreng.	0.10
Monosis volkameriifolia (DC.) H.Rob. & Skvarla	0.10
Morinda angustifolia Roxb.	0.10
Morinda citrifolia L.	0.13
Morinda coreia Buch.-Ham.	0.03
Moringa oleifera Lam.	0.03
Morus alba L.	0.19
Morus macroura Miq.	0.10
Mucuna macrocarpa Wall.	0.13
Mucuna pruriens (L.) DC.	0.16
Muehlenbeckia platyclados (F.Muell.) Meisn.	0.03
Muntingia calabura L.	0.03
Murraya koenigii (L.) Spreng.	0.03
Murraya paniculata (L.) Jack	0.03
Musa × paradisiaca L.	0.26
Musa acuminata Colla	0.23
Mussaenda kerrii Craib	0.03
Mussaenda sanderiana Ridl.	0.52
Myriopteron extensum (Wight) K.Schum.	0.03
Nauclea orientalis (L.) L.	0.13
Nicotiana tabacum L.	0.06
Nyctocalos brunfelsiiflorum Teijsm. & Binn.	0.19
Nymphaea lotus L.	0.03

Ochna integerrima (Lour.) Merr.	0.35
Ocimum americanum L.	0.06
Ocimum basilicum L.	0.03
Ocimum gratissimum L.	0.19
Ocimum tenuiflorum L.	0.13
Ocotea lancifolia (Schott) Mez	0.03
Oenanthe javanica (Blume) DC.	0.06
Ophiopogon yunnanensis S.C.Chen	0.03
Oroxylum indicum (L.) Kurz	0.52
Orthosiphon aristatus (Blume) Miq.	0.13
Oryza sativa L.	0.06
Osbeckia chinensis L.	0.26
Oxalis acetosella L.	0.03
Oxyceros bispinosus (Griff.) Tirveng.	0.03
Paederia foetida L.	0.13
Paederia linearis Hook.f.	0.06
Paederia pallida Craib	0.03
Paederia pilifera Hook.f.	0.06
Pandanus amaryllifolius Roxb. ex Lindl.	0.10
Papaver somniferum L.	0.10
Paris polyphylla Sm.	0.42
Passiflora foetida L.	0.06
Pavetta indica L.	0.10
Pavetta tomentosa Roxb. ex Sm.	0.03
Peliosanthes caesia J.M.H.Shaw	0.16
Peliosanthes macrophylla Wall. ex Baker	0.16
Peliosanthes teta Andrews	0.06
Peperomia pellucida (L.) Kunth	0.16
Persicaria barbata (L.) H.Hara	0.06
Persicaria odorata (Lour.) Soják	0.03
Phlogacanthus curviflorus (Nees) Nees	0.35
Phrynium pubinerve Blume	0.06
Phyllanthus acidus (L.) Skeels	0.10
Phyllanthus amarus Schumach. & Thonn.	0.32
Phyllanthus elegans Wall. ex Müll.Arg.	0.03
Phyllanthus emblica L.	0.48
Phyllanthus sphaerogynus Müll.Arg.	0.03
Phyllodium longipes (Craib) Schindl.	0.06
Phyllodium pulchellum (L.) Desv.	0.26
Physalis angulata L.	0.16
Phytolacca acinosa Roxb.	0.03
Picrasma javanica Blume	0.29
Pinus kesiya Royle ex Gordon	0.10

Piper betle L.	0.29
Piper boehmeriifolium (Miq.) Wall. ex C.DC.	0.06
Piper interruptum Opiz	0.29
Piper nigrum L.	0.10
Piper retrofractum Vahl	0.16
Piper sarmentosum Roxb.	0.16
Pittosporopsis kerrii Craib	0.03
Plantago major L.	0.81
Platycerium wallichii Hook.	0.19
Plumbago indica L.	0.45
Plumbago zeylanica L.	0.26
Plumeria obtusa L.	0.29
Plumeria rubra L.	0.13
Poikilospermum suaveolens (Blume) Merr.	0.03
Polygala arillata Buch.-Ham. ex D.Don	0.23
Polygala chinensis L.	0.19
Polygala crotalarioides Buch.-Ham. ex DC.	0.03
Polygonum paleaceum Wall. ex Hook.f.	0.06
Polyscias filicifolia (C.Moore ex E.Fourn.) L.H.Bailey	0.03
Pontederia crassipes Mart.	0.03
Pothos chinensis (Raf.) Merr.	0.45
Pothos scandens L.	0.74
Praxelis clematidea (Hieron. ex Kuntze) R.M.King & H.Rob.	0.06
Protium serratum (Wall. ex Colebr.) Engl.	0.06
Prunus cerasoides Buch.-Ham. ex D.Don	0.10
Pseuderanthemum graciliflorum (Nees) Ridl.	0.10
Psidium guajava L.	0.77
Psophocarpus tetragonolobus (L.) DC.	0.03
Psychotria yunnanensis Hutch.	0.10
Pteridium aquilinum (L.) Kuhn	0.10
Pteridrys syrmatica (Willd.) C.Chr. & Ching	0.06
Pterocarpus macrocarpus Kurz	0.06
Pueraria candollei Wall. ex Benth.	0.06
Punica granatum L.	0.16
Putranjiva roxburghii Wall.	0.06
Raphanus raphanistrum L.	0.03
Rauvolfia serpentina (L.) Benth. ex Kurz	0.10
Rauvolfia verticillata (Lour.) Baill.	0.16
Rhinacanthus nasutus (L.) Kurz	0.13
Ricinus communis L.	1.00
Rotheca serrata (L.) Steane & Mabb.	0.87
Rubia cordifolia L.	0.16
Rubus alceifolius Poir.	0.03

Rubus ellipticus Sm.	0.03
Ruellia tuberosa L.	0.03
Saccharum officinarum L.	0.23
Salacia chinensis L.	0.06
Salacia verrucosa Wight	0.29
Salix tetrasperma Roxb.	0.06
Sambucus canadensis L.	0.48
Sambucus javanica Reinw. ex Blume	1.35
Sapindus rarak DC.	0.10
Sarcandra glabra (Thunb.) Nakai	0.16
Sarcosperma arboreum Hook.f.	0.03
Saurauia napaulensis DC.	0.06
Saurauia roxburghii Wall.	0.03
Scadoxus multiflorus (Martyn) Raf.	0.03
Scaphium macropodum (Miq.) Beumée ex K.Heyne	0.03
Schefflera bengalensis Gamble	0.10
Schefflera leucantha R.Vig.	0.45
Schefflera venulosa (Wight & Arn.) Harms	0.03
Schima wallichii (DC.) Korth.	0.84
Schleichera oleosa (Lour.) Oken	0.03
Scleropyrum maingayi Hook.f.	0.03
Scleropyrum pentandrum (Dennst.) Mabb.	0.39
Scoparia dulcis L.	1.10
Scurrula ferruginea (Roxb. ex Jack) Danser	0.10
Scutellaria indica L.	0.03
Selliguea cruciformis (Ching) Fraser-Jenk.	0.06
Senegalia caesia (L.) Maslin, Seigler & Ebinger	0.16
Senegalia catechu (L.f.) P.J.H.Hurter & Mabb.	0.03
Senegalia rugata (Lam.) Britton & Rose	0.13
Senna alata (L.) Roxb.	0.77
Senna hirsuta (L.) H.S.Irwin & Barneby	0.06
Senna occidentalis (L.) Link	0.32
Senna siamea (Lam.) H.S.Irwin & Barneby	0.10
Senna timoriensis (DC.) H.S.Irwin & Barneby	0.03
Senna tora (L.) Roxb.	0.13
Sesamum indicum L.	0.06
Setaria parviflora (Poir.) Kerguélen	0.03
Shorea obtusa Wall. ex Blume	0.03
Shorea roxburghii G.Don	0.06
Sida acuta Burm.f.	0.61
Sida cordifolia L.	0.10
Sida rhombifolia L.	0.19
Smilax blumei A.DC.	0.03

Smilax corbularia Kunth	0.35
Smilax glabra Roxb.	0.32
Smilax griffithii A.DC.	0.06
Smilax luzonensis C.Presl	0.13
Smilax ovalifolia Roxb. ex D.Don	0.32
Smilax zeylanica L.	0.03
Sohmaea teres (Wall. ex Benth.) H.Ohashi & K.Ohashi	0.03
Solanum aculeatissimum Jacq.	0.03
Solanum americanum Mill.	0.13
Solanum erianthum D.Don	0.06
Solanum lasiocarpum Dunal	0.06
Solanum lycopersicum L.	0.06
Solanum nigrum L.	0.03
Solanum torvum Sw.	0.06
Solanum trilobatum L.	0.03
Solanum violaceum Ortega	0.32
Solanum virginianum L.	0.06
Spatholobus parviflorus (Roxb. ex G.Don) Kuntze	0.13
Spondias bipinnata Airy Shaw & Forman	0.03
Spondias pinnata (L.f.) Kurz	0.10
Stachytarpheta indica (L.) Vahl	0.03
Stachytarpheta jamaicensis (L.) Vahl	0.16
Stephania japonica (Thunb.) Miers	0.13
Stephania pierrei Diels	0.06
Stereospermum neuranthum Kurz	0.03
Streblus asper Lour.	0.13
Streptocaulon juventas (Lour.) Merr.	0.06
Strobilanthes cusia (Nees) Kuntze	0.26
Strobocalyx arborea (Buch.-Ham.) Sch.Bip.	0.03
Strobocalyx solanifolia Sch.Bip.	0.03
Strychnos nux-vomica L.	0.06
Styrax benzoides Craib	0.10
Sumbaviopsis albicans (Blume) J.J.Sm.	0.03
Suregada multiflora (A.Juss.) Baill.	0.03
Symphorema involucratum Roxb.	0.13
Syzygium cumini (L.) Skeels	0.19
Tabernaemontana divaricata (L.) R.Br. ex Roem. & Schult.	0.06
Tabernaemontana pandacaqui Poir.	0.03
Tacca chantrieri André	0.06
Tadehagi triquetrum (L.) H.Ohashi	0.81
Tagetes erecta L.	0.03
Tamarindus indica L.	0.23
Tarennoidea wallichii (Hook.f.) Tirveng. & Sastre	0.03

Tectaria polymorpha (Wall. ex Hook.) Copel.	0.06
Tectona grandis L.f.	0.06
Terminalia bellirica (Gaertn.) Roxb.	0.10
Terminalia chebula Retz.	0.13
Tetrastigma cruciatum Craib & Gagnep.	0.06
Thunbergia coccinea Wall. ex D.Don	0.39
Thunbergia laurifolia Lindl.	1.10
Thyrsostachys siamensis Gamble	0.06
Thysanolaena latifolia (Roxb. ex Hornem.) Honda	0.19
Tiliacora triandra (Colebr.) Diels	0.03
Tinomiscium petiolare Hook. f. & Thomson	0.10
Tinospora baenzigeri Forman	0.03
Tinospora crispa (L.) Hook. f. & Thomson	1.48
Tinospora sinensis (Lour.) Merr.	0.13
Tithonia diversifolia (Hemsl.) A.Gray	0.32
Toddalia asiatica (L.) Lam.	0.10
Toona hexandra (Wall.) M.Roem.	0.03
Torenia fournieri Linden ex E.Fourn.	0.03
Tradescantia spathacea Sw.	0.03
Trema orientale (L.) Blume	0.03
Trevesia palmata (Roxb. ex Lindl.) Vis.	0.13
Triadica cochinchinensis Lour.	0.10
Trichosanthes scabra Lour.	0.06
Trichosanthes tricuspidata Lour.	0.06
Tridax procumbens L.	0.03
Tristaniopsis burmanica (Griff.) Peter G.Wilson & J.T.Waterh.	0.06
Tropidia angulosa (Lindl.) Blume	0.03
Tupistra muricata (Gagnep.) N.Tanaka	0.06
Typhonium trilobatum (L.) Schott	0.03
Uncaria laevigata Wall. ex G.Don	0.06
Uraria cordifolia Wall.	0.03
Urena lobata L.	0.03
Uvaria siamensis (Scheff.) L.L.Zhou, Y.C.F.Su & R.M.K.Saunders	0.03
Vaccinium sprengelii (G.Don) Sleumer ex Rehder	0.16
Ventilago denticulata Willd.	0.19
Ventilago harmandiana Pierre	0.06
Verbena officinalis L.	0.19
Viburnum sambucinum Reinw. ex Blume	0.03
Vigna dalzelliana (Kuntze) Verdc.	0.03
Viscum articulatum Burm.f.	0.06
Vitex peduncularis Wall. ex Schauer	0.16
Vitex trifolia L.	0.23
Walsura robusta Roxb.	0.03

Wrightia arborea (Dennst.) Mabb.	0.03
Wurfbainia testacea (Ridl.) Skornick. & A.D.Poulsen	0.03
Wurfbainia villosa (Lour.) Skornick. & A.D.Poulsen	0.03
Xantolis cambodiana (Pierre ex Dubard) P.Royen	0.42
Xylia xylocarpa (Roxb.) W.Theob.	0.10
Zanthoxylum rhetsa (Roxb.) DC.	0.03
Zephyranthes candida (Lindl.) Herb.	0.03
Zephyranthes rosea Lindl.	0.06
Zingiber latifolium Theilade & Mood	0.06
Zingiber montanum (J.Koenig) Link ex A.Dietr.	0.52
Zingiber officinale Roscoe	0.16
Zingiber ottensii Valeton	1.10
Zingiber rubens Roxb.	0.03
Zingiber zerumbet (L.) Roscoe ex Sm.	0.13
Ziziphus cambodiana Pierre	0.32

Table S2 Number of use reports for each symptom/treatment/uses of the top ten most used plant species by Karen in Thailand

**Symptoms/treatments/uses**	***Biancaea sappan***	***Chromolaena odorata***	***Elephantopus scaber***	***Mimosa pigra***	***Ricinus communis***	***Sambucus javanica***	***Scoparia dulcis***	***Thunbergia laurifolia***	***Tinospora crispa***	***Zingiber ottensii***
Abdominal pain	1									5
Abscess							1			
Abstergent							1			
Alcohol detoxication								2		
Amniotic fluid elimination	2									
Analeptic			1						1	
Anthelminthics									3	
Antidote		1					1	2		
Anuria		1	4	4		1				
Aphrodisiac						1				
Appetite stimulant	1								5	
Asthma	1	1						1		
Athlete's foot		1					1			
Back and waist pain	4	1	1							
Bloat								1	1	1
Blood pressure							1			
Blood tonic	6			2					2	
Body recovery from sickness			1	1						

Bone fractures		1			1	9				
Bruises						1				
Burns						4	3			
Cancer									1	
Carminative										1
Catepillar allergy										1
Chest pain									1	
Chopped lips			1							
Cold			2				1	1	1	
Colitis	1									
Conjuctivitis								1		
Constipation				1						1
Cough	1	1	4			1	2		1	
Dermatosis							1			
Detoxicant								1		
Diabetes									3	
Diarrhea	2	1	1			1	1	1		3
Dizziness	1	1			5			1		1
Dysentery	1									
Dysmenorrhea	4									
Earache					1					
Epilepsy		1								
Epistaxis		1								
Expectorant			1							
Eye drops								1		
Eye irritation								1		
Eye pain								1		
Faintness							1			
Fever	1	2	3	6		1	3	2	2	
Flatulence									2	6
Food poisoning			1		1					
Gall stones			1	1						
Gastritis	1	1							1	
Haemostasis		5								
Hangover								1		
Headache					2				1	
Hemorrhoids				2						
Hidrotic			1							
Hypertension								1	1	
Indigestion			1		1					
Inflamation					1	1				
Insect bites								1		
Internal body heat								1		

Intoxication								2		
Irregular menstruation	2				5	1				1
Jaundice	1		1							
Joint pain						1				
Kidney detoxification			1						1	
Lactagogue			1							
Laxative					1					5
Leukorrhea				1						
Lung tonic	1									
Malaria		1	1			1	1		2	
Muscular pain		3	3	4	1	5	2	2	3	2
Muscular relaxant	3		1		2	2		1		
Oedema			1	4		2	1			
Otitis					2					
Otorrhea					1					
Pain						1				
Palpitation		1								
Peptic ulcers		2							1	
Phlegm	1									
Pneumonia									1	
Poisonous insect bites								2		
Post partum recovery										1
Postpartum muscular relaxation	1									
Postpartum nervous	1	1			1					
Postpartum tonic	2				1	1				4
Pruritus		1			1				1	
Puerperium	1									
Pus	1									
Rheumatoid arthritis						1				
Snake bites								4		
Sore throat							1			
Sprain						4				
Stomach ache					1		1			1
Stop bleeding		14								
Thirsty	1									
Tick bites									2	
Tinnitus					1					
Tonic	5		5			1	1		5	
Tootache			1					2		
Tuberculosis	1									
Typhoid									3	
Umbilical wounds										1

Urinary stones		1	1	4	1		1			
Venomous snake bites								1		
Wounds	1	7			1	2	9		1	
Total	49	50	39	30	31	42	34	34	46	34

Table S3 CI values of medicinal plants families used by Karen in Thailand

Fam	CI
Acanthaceae	3.32
Acoraceae	0.77
Actinidiaceae	0.10
Adoxaceae	0.48
Alismataceae	0.03
Amaranthaceae	0.42
Amaryllidaceae	0.45
Anacardiaceae	0.45
Annonaceae	1.29
Apiaceae	0.87
Apocynaceae	2.68
Aquifoliaceae	0.03
Araceae	1.32
Araliaceae	1.13
Arecaceae	0.19
Aristolochiaceae	0.13
Asparagaceae	1.52
Aspleniaceae	0.10
Asteraceae	7.13
Athyriaceae	0.06
Balsaminaceae	0.06
Basellaceae	0.19
Berberidaceae	0.03
Betulaceae	0.68
Bignoniaceae	1.48
Bixaceae	0.03
Boraginaceae	0.10
Brassicaceae	0.03
Bromeliaceae	0.03
Burseraceae	0.10
Buxaceae	0.03
Campanulaceae	0.16
Cannabaceae	0.48
Cannaceae	0.23
Capparaceae	0.19
Cardiopteridaceae	0.06

Caricaceae	0.10
Caryophyllaceae	0.10
Celastraceae	0.55
Chloranthaceae	0.84
Clusiaceae	0.06
Colchicaceae	0.03
Combretaceae	0.58
Commelinaceae	0.10
Connaraceae	0.10
Convolvulaceae	0.39
Costaceae	0.58
Crassulaceae	0.16
Cucurbitaceae	0.58
Cycadaceae	0.03
Cyperaceae	0.13
Dennstaedtiaceae	0.16
Dilleniaceae	0.10
Dioscoreaceae	0.19
Dipterocarpaceae	0.23
Ebenaceae	0.26
Elaeagnaceae	0.03
Equisetaceae	0.55
Ericaceae	0.23
Euphorbiaceae	4.39
Gentianaceae	0.06
Gnetaceae	0.10
Hernandiaceae	0.19
Hypericaceae	0.48
Hypoxidaceae	0.29
Icacinaceae	0.03
Iridaceae	0.48
Juglandaceae	0.13
Lamiaceae	4.35
Lauraceae	1.84
Lecythidaceae	0.16
Leguminosae	11.87
Liliaceae	0.06
Linderniaceae	0.03
Loganiaceae	0.06
Loranthaceae	0.29
Lycopodiaceae	0.06
Lygodiaceae	0.45
Lythraceae	0.48

Magnoliaceae	0.03
Malpighiaceae	0.35
Malvaceae	1.68
Marantaceae	0.16
Marattiaceae	0.32
Martyniaceae	0.06
Melanthiaceae	0.42
Melastomataceae	0.74
Meliaceae	0.65
Menispermaceae	2.68
Molluginaceae	0.03
Moraceae	1.48
Moringaceae	0.03
Muntingiaceae	0.03
Musaceae	0.65
Myristicaceae	0.03
Myrsinaceae	0.10
Myrtaceae	1.03
Nyctaginaceae	0.03
Nymphaeaceae	0.03
Ochnaceae	0.35
Olacaceae	0.03
Oleaceae	0.32
Onagraceae	0.03
Orchidaceae	0.45
Orobanchaceae	0.10
Oxalidaceae	0.19
Pandanaceae	0.10
Papaveraceae	0.23
Passifloraceae	0.10
Pedaliaceae	0.06
Phyllanthaceae	2.06
Phytolaccaceae	0.03
Pinaceae	0.10
Piperaceae	1.23
Plantaginaceae	2.00
Plumbaginaceae	0.71
Poaceae	2.45
Polygalaceae	0.45
Polygonaceae	0.19
Polypodiaceae	0.45
Pontederiaceae	0.03
Primulaceae	0.58

Proteaceae	0.29
Putranjivaceae	0.06
Ranunculaceae	0.16
Rhamnaceae	0.68
Rosaceae	0.16
Rubiaceae	2.39
Rutaceae	2.23
Salicaceae	0.48
Santalaceae	0.71
Sapindaceae	0.58
Sapotaceae	0.45
Saururaceae	0.23
Scrophulariaceae	0.39
Simaroubaceae	0.65
Smilacaceae	1.26
Solanaceae	1.23
Staphyleaceae	0.16
Styracaceae	0.10
Taccaceae	0.03
Tectariaceae	0.06
Theaceae	0.97
Thymelaeaceae	0.06
Urticaceae	0.06
Verbenaceae	0.48
Viburnaceae	1.39
Vitaceae	1.06
Xanthorrhoeaceae	0.35
Zingiberaceae	5.06
